# Impact of the Tumor Microenvironment and Molecular Oncology in Peritoneal Metastases

**DOI:** 10.3390/cancers18132143

**Published:** 2026-07-03

**Authors:** Abaan Khurshid, Haarika S. Chalasani, Anna Jacobs, Joao Pedro Kasakewitch, Kevin Avila, Zachary J. Brown

**Affiliations:** 1 Department of Surgery, NYU Grossman Long Island School of Medicine, Mineola, NY 11501, USA; abaan.khurshid@nyulangone.org (A.K.); joaopedro.gkasakewitch@nyulangone.org (J.P.K.); 2 Department of Surgery, Division of Surgical Oncology, NYU Langone Health, NYU Grossman Long Island School of Medicine, Mineola, NY 11501, USA

**Keywords:** HIPEC, peritoneal, carcinomatosis, intraperitoneal chemotherapy, cytoreductive surgery, tumor microenvironment, molecular oncology

## Abstract

Background/Objectives: Peritoneal metastases (PMs) arise from gastrointestinal, gynecologic, hepatobiliary, and colorectal origins and are associated with poor outcomes. Cytoreductive surgery (CRS) with intraperitoneal (IP) chemotherapy offers benefit for select patients, but survival remains limited. This review aims to summarize recent insights into the molecular and tumor microenvironmental (TME) changes characteristic of PMs and the impact of IP chemotherapy. Methods: A literature review was performed using recent clinical, translational, and preclinical studies examining alterations in molecular signaling, DNA repair alterations, metabolic pathways, and angiogenic factors in PMs before and after IP therapy. Results: Peritoneal metastases exhibit distinct biology after being treated with IP chemotherapy. Treatment induces alterations in gene expression, mutational patterns, and immune infiltrates. Heated intraperitoneal chemotherapy (HIPEC) has been associated with increased CD8+ T-cell activity, macrophage and NK cell shifts, and modulation of PD-1/PD-L1 signaling, which correlate with treatment response and survival. Emerging data on PIPAC similarly suggests induction of favorable gene expression changes with repeated treatment, though supporting evidence remains more limited than for HIPEC. Angiogenic pathways—particularly VEGF and HIF1α—remain key drivers of PM progression and predictors of post-operative outcomes. Early findings suggest potential synergy between IP chemotherapy and immunotherapy though clinical trials are ongoing. Conclusions: IP chemotherapy induces tumor microenvironmental changes that have potential to shape therapeutic response. Characterizing these measurable biologic changes may allow clinicians to improve patient selection and support the development of combination therapies to enhance outcomes.

## Simple Summary

Peritoneal metastases (PMs) represent a devastating diagnosis that can occur from a wide variety of malignancies. Advances have been made in the management of PMs, particularly utilizing cytoreductive surgery (CRS) and intraperitoneal (IP) chemotherapy. Ongoing research is exploring the utility of various IP chemotherapeutic agents, various modalities of IP chemotherapy delivery, and conjunctive use of immunologic agents. As such, it is essential to understand the molecular alterations and tumor microenvironmental changes that occur in PMs and following IP chemotherapy. In this review, we will highlight some recent key findings regarding the molecular oncology and tumor microenvironment of peritoneal metastases.

## 1. Introduction

Peritoneal metastases (PMs) occur from a wide variety of malignancies of gastrointestinal, gynecological, hepatopancreatobiliary, and primary peritoneal origin. The biological basis behind the development of PM is multifactorial. Key steps include shedding of tumor cells from the primary tumor site, transport within the abdomen directed by several biomechanical factors (including gravity, respiratory mechanics, and bowel peristalsis), and attachment with eventual invasion into the mesothelial lining of the abdomen [1]. A unique factor for the development of PM is the mechanics of locoregional intra-abdominal fluid flow that directs flow of the circulating tumor cells in the abdomen; this is in contrast to hematogenous metastatic pathways which allow for seeding of tumor cells in distant organs. The tumor microenvironment is critical to understanding pathogenesis behind PM, as it is through specific tissue ecosystems that tumor cells circulating throughout the abdomen can attach and invade to form metastases. For example, the immune-rich omentum, when prompted by pro-metastatic inflammatory factors, is stimulated to express neutrophil extracellular traps which act as a favorable ecosystem for direct attachment and invasion of circulating tumor cells [1,2]. In addition to immune environment factors, the extracellular matrix (ECM) has also been shown to play a key role with circulating cancer cell adhesion to ECM collagen in a pro-metastatic niche leading to downstream mesothelial invasion and the development of PM. Cell-to-cell adhesion mechanisms supported by fibroblasts have been shown to be important for the development of tumor cell aggregates that can invade the peritoneum to form PM [3]. Beyond biochemical adhesion, the biomechanical properties of the ECM are increasingly recognized as drivers of PM pathogenesis; increased peritoneal tissue stiffness promotes mechanotransduction signaling (e.g., YAP/TAZ activation) in tumor and stromal cells, reinforcing a fibrotic, pro-invasive niche that favors tumor cell attachment and CAF activation [4]. Thus, the importance of the tumor microenvironment in peritoneal metastasis is clear, as the development of PM is the result of diverse interactions between tumor cells, surrounding immune cells, and ECM components.

PMs are often a devastating diagnosis for patients and providers with limited treatment options. For select patients, radical cytoreductive surgery (CRS) with complete extirpation of the tumor with or without intraperitoneal (IP) chemotherapy has demonstrated a survival advantage [5]. Although systemic chemotherapy is the cornerstone of treatment for patients with metastatic disease, patients with PMs often have a worse prognosis than other metastatic disease sites, such as the liver or lung, which is often attributed to poor vascularity of the peritoneum and limited tumor penetration of systemic chemotherapy [6]. This is in contrast to liver metastases which typically arise via hematogenous dissemination through the portal venous system into a single, highly vascularized organ, permitting more effective systemic drug delivery and access to ablative or locoregional therapies that are less readily applied to the diffusely distributed, poorly perfused peritoneal surface.

The magnitude of clinical benefit conferred by IP chemotherapy varies considerably by tumor origin. In colorectal PM, the phase 3 PRODIGE 7 trial found that adding oxaliplatin-based HIPEC to complete CRS did not improve overall survival compared with CRS alone (median OS 41.7 vs. 41.2 months; HR 1.00; *p* = 0.99), prompting a reassessment of HIPEC’s role in this population and shifting emphasis toward complete cytoreduction itself as the primary determinant of outcome [1]. In contrast, the phase 3 OVHIPEC-1 trial demonstrated a clear survival benefit of cisplatin-based HIPEC added to interval CRS in stage III epithelial ovarian cancer, with improved recurrence-free survival (14.3 vs. 10.7 months; HR 0.63; *p* = 0.0008) and overall survival (44.9 vs. 33.3 months; HR 0.70; *p* = 0.011) that was sustained at 10-year follow-up [7]. In gastric cancer with peritoneal metastasis, the phase 3 PHOENIX-GC trial comparing intraperitoneal plus systemic paclitaxel against systemic cisplatin/S-1 did not meet its primary endpoint for median OS (17.7 vs. 15.2 months; HR 0.72; *p* = 0.080), though the IP arm showed a substantially higher 3-year OS rate (21.9% vs. 6.0%); a more recent phase 3 trial of neoadjuvant intraperitoneal paclitaxel reported a statistically significant OS benefit (19.4 vs. 13.9 months; HR 0.67; *p* = 0.01) [8,9]. For appendiceal-origin PM, iterative HIPEC has been associated with favorable mutational and tumor-immune microenvironment changes linked to progression-free survival, though randomized outcome data remain more limited than for colorectal or ovarian disease [10]. These tumor-type-specific differences underscore that the benefit of IP chemotherapy cannot be generalized across peritoneal surface malignancies and instead depends on underlying tumor biology, chemosensitivity, and trial design.

**Figure 1:**
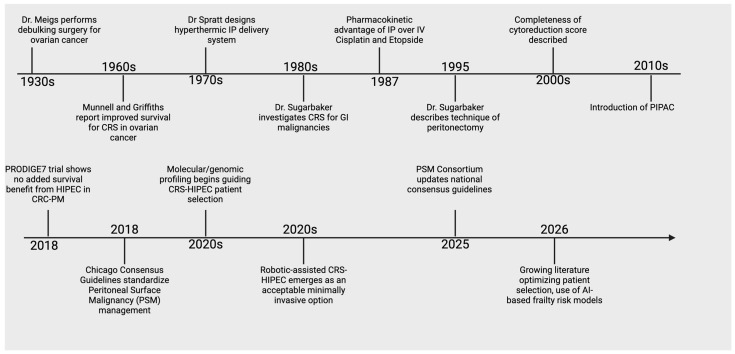
Cytoreductive surgery and intraperitoneal chemotherapy is not a new concept with radical tumor debulking occurring in the 1930s for ovarian cancer. Since that time, significant advancements have been made in surgical technique and intraperitoneal chemotherapy delivery. CRS, cytoreductive surgery; IP, intraperitoneal; GI, gastrointestinal; IV, intravenous; PIPAC, pressurized intraperitoneal aerosolized chemotherapy.

Surgical resection of PMs is not a new concept and was first described by Dr. Meigs in the 1930s for tumor debulking of metastatic ovarian cancer [11]. Since that time, advances have been made with CRS for gastrointestinal malignancies and the introduction of heated intraperitoneal chemotherapy (HIPEC) (Figure 1). Other methods of IP chemotherapy have been explored including normothermic intraperitoneal chemotherapy (NIPEC)/early post-operative chemotherapy (EPIC) often with administration of chemotherapy through a subcutaneous catheter tunneled into the abdomen [12] (Table 1).

Pressurized intraperitoneal aerosolized chemotherapy (PIPAC), a more novel method of IP chemotherapy, was developed to deliver chemotherapy laparoscopically as an aerosol under standard laparoscopic pressure [13]. Animal models utilizing methylene blue have demonstrated PIPAC increased penetrance, while pneumoperitoneum allows for aerosolized chemotherapeutic agents to better distribute within the abdomen [14,15].

Though a variety of IP instillation methods exist, HIPEC remains the most utilized technique. The effects of IP chemotherapy on tumor response have been evaluated but are still poorly understood. To better understand the benefits of IP chemotherapy, it is crucial to understand the molecular changes and alterations of the tumor microenvironment (TME) after treatment. In this review, we highlight key findings with relation to the molecular changes and alterations in the TME of PMs as well as the impact of IP chemotherapy.

## 2. Molecular Alterations

### 2.1. Phenotypic Transition

**Figure 2:**
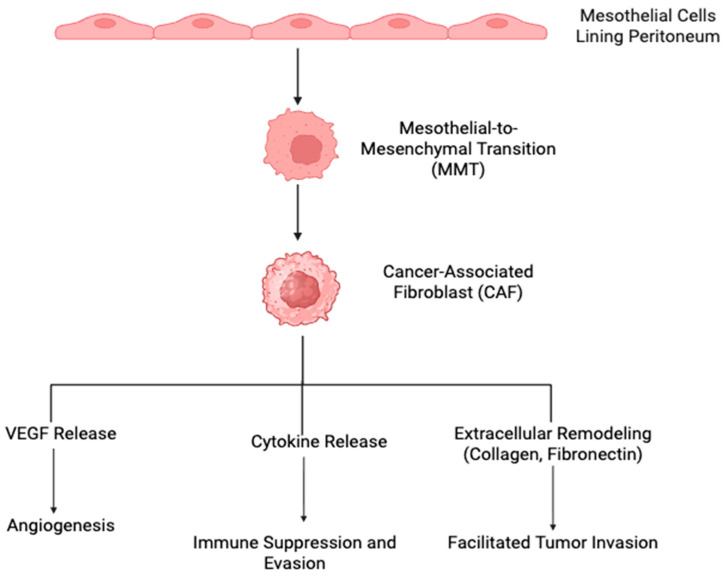
A model demonstrating phenotypical transformation as discussed within this section. Normal peritoneal lining is composed of a single layer of mesothelial cells which can transform into cells with invasive potential, a process termed mesothelial-to-mesenchymal transition. Subsequently, activated stromal fibroblasts (CAFs) aid in the progression of PM by prompting angiogenesis, immune evasion, and overall tumor invasion.

The normal peritoneal lining is characterized by a single layer of mesothelial cells that forms a semipermeable membrane [16]. The transition of these normal mesothelial cells to cells with invasive potential is termed mesothelial-to-mesenchymal transition (MMT) [17]. This process is multifactorial, with several local drivers including cytokines (IL-8) and growth factors (e.g., TGF-B1, TNF-a, VEGF, HGF) in peritoneal fluid stimulating molecular mechanisms of the transition (Figure 2) [10,18]. These environmental drivers in turn stimulate transcription regulators, such as snail, which has been implicated as a mediator of MMT through downregulation of E-cadherin expression and disruption of cell-to-cell adhesion of the normal mesothelial lining [17]. Additional key factors of the transition include the loss of cell polarity, cytoskeletal reorganization, matrix metalloprotease upregulation, and basement membrane degradation which all contribute to the invasive potential of cells undergoing MMT [16].

Interrelated with MMT in PM is the stromal environment which allows for stimulation of growth and invasive potential. Cancer-associated fibroblasts (CAFs), or activated stromal fibroblasts, play a role in stimulating MMT as well as angiogenesis to support PM growth [18]. MMT and activation of CAFs appear to be synergistic, where a portion of CAFs have been shown to be derived from mesothelial cells and lead to improved attachment and invasion of circulating tumor cells. As tumor implants grow, CAFs are recruited from the nearby mesothelial tissue and propagate continued tumor progression [19].

Molecular differences exist between the primary tumor and metastatic disease. Although primary tumors shed a large number of tumor cells, only a fraction will develop into metastases as metastatic development depends on the acquisition of traits that promote survival of tumor cells as well as premetastatic niche reprogramming [20,21]. Mechanisms behind mesothelial cell conversion, tumor cell adhesion and invasion, and the role of the microenvironment as a mediator is an active area of ongoing research as an exciting target for therapeutic strategies.

### 2.2. Peritoneal Metastatic Niche

Development of the peritoneal metastatic niche is not a discrete process, but rather a combination of diverse mechanisms. Exosomes, or extracellular vesicles, are secreted by cells and carry bioactive proteins, genetic material, and metabolites that act as a form of inter-cellular communication in peritoneal metastatic niche development. Tumor cell-derived exosomes are critical for priming the peritoneum in PM [22]. For example, Yokoi et al. identified *MMP1* (matrix metalloproteinase-1) mRNA in exosomes derived from a high-grade ovarian cancer cell line as well as from patient ascites and this was associated with peritoneal metastasis [23]. It has also been shown that naturally secreted tumor cell exosomes can differ from exosomes secreted in response to an extracellular stimulus. Calcium chelation-induced exosomes in epithelial ovarian cancer have been shown to contain different miRNA signatures compared to non-stimulated tumor cell-derived exosomes, with stimulated exosomes playing a role in rapid tumor cell adhesion and proliferation and non-stimulated exosomes playing a role in mesenchymal transition [24]. Another study identified that ovarian cancer-derived exosomal ANXA2 interacting with toll-like receptor-2 on human peritoneal mesothelial cells promoted MMT [25]. In gastric cancer, numerous exosomal cargos have been implicated in stimulating MMT, immune regulation, angiogenesis, and conversion of stromal cells into CAFs [19,26,27,28,29,30]. Chen et al. compared patients with primary GC and GC PMs. Compared to primary GC, PM samples were enriched in extracellular exosomes and cell adhesion pathways [31]. Remarkable heterogeneity exists within possible contents of exosomes, and through a diverse array of mechanisms they allow for crosstalk between tumor cells, mesothelial cells, and stroma which is critical for the development of PM and is an active area of biomarker research.

In addition to inter-cellular communication via exosomes, transcriptional pathways play a major role in the development of PM. Assessment of ovarian high-grade serous carcinoma tumor samples from 160 patients showed epigenetic contribution to peritoneal metastasis, with reduced methylation at promotors of genes associated with stromal remodeling and MMT [32]. Similarly, Barriuso et al. identified 20 genes with altered regulation in PMs from colorectal cancer primary that promote a “stemness” phenotype along with pro-tumor inflammatory changes indicating adaptation of the peritoneal niche through genes such as CD36, IL6, MMP1, and CLCA4 [33].

Metabolic reprogramming also plays a role in the development of the peritoneal metastatic niche. In colorectal PM, CAFs have been shown to upregulate CPT1A (carnitine palmitoyltransferase IA), a rate-limiting step of fatty acid oxidation, to allow for increased oxidation and minimal glycolysis. As an intriguing target for therapy, inhibition of fatty acid oxidation in CAFs decreased peritoneal metastatic dissemination [34]. Transcriptional assessment of tumor samples from patients with ovarian high-grade serous carcinoma also revealed upregulation of lipid mobilization pathways that indicated preference for beta-oxidation pathways [32]. GC cells have been found to secrete laminin gamma 1 (LAMC1) and is highly expressed in PMs. LAMC1 promoted preadipocytes to mature, rupture and release free fatty acids (FFAs) in the peritoneal TME to form the premetastatic niche [35].

The immune composition of the peritoneal environment is also closely related to the development of PM. Lymphoid follicles in the omentum, termed milky spots, are a prime environment for tumor cell attachment through increased expression of cell adhesion mediators such as ICAM-1 and VCAM-1. Tissue-resident macrophages within milky spots also play a role in immunosuppression [36]. It has also been shown that peritoneal resident macrophages within the peritoneal ecosystem highly express immunosuppressive genes such as APOE, FOLR2, and MRC2, amongst others, and allow for immune evasion of tumor cells [37]. Immune reorganization is key for a tumor cell’s ability to attach and survive in the peritoneal metastatic niche to progress towards invasive PM.

**Figure 3:**
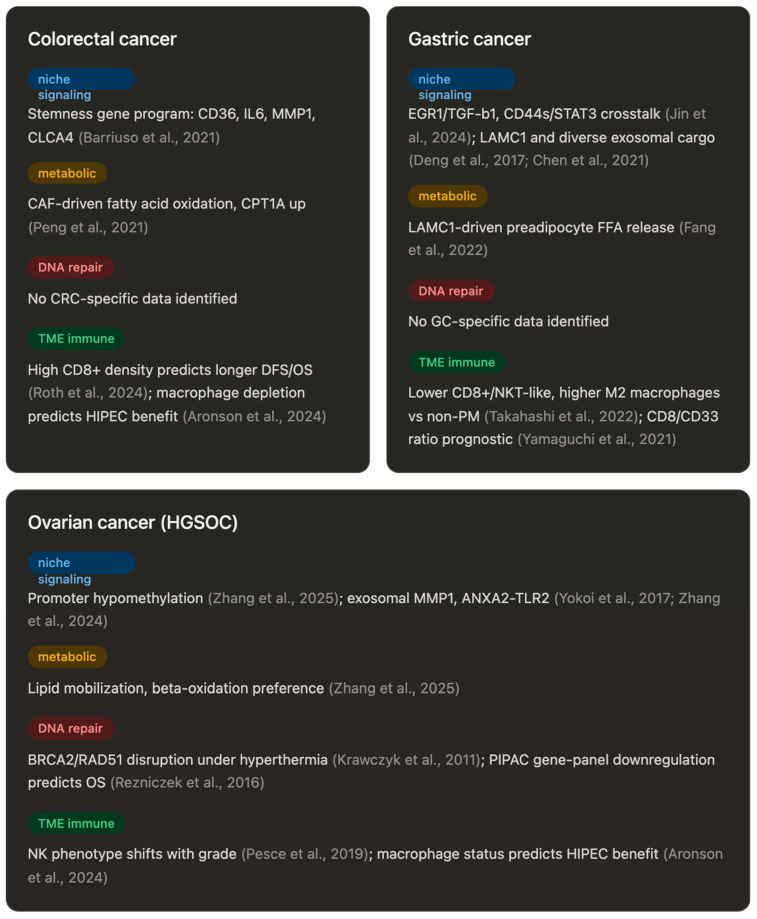
Tumor type-specific molecular and microenvironmental features of peritoneal metastasis. Niche signaling, metabolic reprogramming, DNA repair alterations, and tumor microenvironment (TME)/immune characteristics are summarized for colorectal, gastric, and ovarian (HGSOC) carcinomas with peritoneal spread, highlighting mechanistic heterogeneity across primary tumor types [23,25,26,29,30,32,33,34,35,38,39,40,41,42,43,44].

Collectively, these findings indicate that metabolic remodeling in PM is highly context-dependent, shaped by dynamic interactions between tumor cells and stromal/adipose components rather than a single conserved pathway (Figure 3). Development of the peritoneal metastatic niche is a heterogenous process involving interplay between tumor cells, mesothelial cells, immune cells, and stroma to prime the peritoneum to accept and support metastasis. This is an active area of ongoing research for both therapeutic and, interestingly, preventative interventions.

### 2.3. Molecular Alterations in Response to Treatment

DNA repair and its regulation are important not only in the progression of cancer, but its response to treatment. DNA repair mechanisms are triggered in response to damaging agents, and failure to maintain genomic homeostasis can lead to proliferation of dysplastic and malignant cell lines [45]. Molecular alterations have been investigated after IP chemotherapy. IP chemotherapy has been shown to alter DNA repair regulation. The breast cancer susceptibility gene (BRCA) is an important tumor suppressor gene that maintains genomic stability by repairing double-strand breaks [46]. Studies have shown that hyperthermia between 41 and 42.5 degrees Celsius can induce degradation of BRCA2 and inhibit homologous recombination in DNA repair [38]. Specifically, heat-induced degradation of BRCA2 results in reduced RAD51 focus formation causing this homologous recombination inhibition. This correlation has been shown to be dose-dependent; as temperatures continue to rise, there is a positive correlation with cell death. However, with increasing hyperthermia, cells die directly from other heat-related processes as opposed to homologous recombination defects. Hyperthermia has also been implicated in damaging various other DNA repair genes including ATM, SMC, ATR, and nucleotide excision repair [47]. In vivo, Dellingeret al. demonstrated downregulation of DNA repair pathways in patients with ovarian cancer with peritoneal metastases pre- and post-HIPEC [48]. Additionally, gene expression alterations have been investigated during PIPAC. Rezniczek et al. studied gene expression changes in peritoneal biopsies in patients who underwent iterative PIPAC for peritoneal carcinomatosis. Most patients had primary ovarian cancer (58/63 patients). Tumor samples prior to PIPAC treatment had no signatures predicting eventual clinical response. However, changes in gene expression patterns after repeated PIPAC procedures were prognostic of overall survival (OS), with downregulation of the whole gene panel during IP chemotherapy being associated with better treatment response and survival [39]. Research into molecular changes induced by intraperitoneal chemotherapy administration, and optimization of an anti-tumor effect, is the focus of several clinical trials.

## 3. Angiogenesis

Angiogenesis, the formation of new blood vessels, is key in the development of a receptive microenvironment for tumor metastasis. Metastatic tumor cells have been found to migrate toward the most approximate microvessel [49]. The peritoneum is perfused with blood capillaries and a few arterioles and venules found in the submesothelial layer [50]. The total surface area of the peritoneum is large while the total blood flow to the peritoneum only represents a small fraction of cardiac output [51,52,53]. PMs can range in a variety of sizes with many tumors being only a few millimeters and not appreciated on abdominal imaging [54]. In small tumors less than 1 mm, there is often no vessel formation and tumors rely on permeation for nutrients [55]. In order for tumors to grow larger than a few millimeters, peritoneal metastasis must acquire an additional supply of nutrition and oxygen through a blood vessel network [51]. Li et al. have shown that tumor hypoxia is closely associated with blood perfusion where microscopic tumors were avascular and hypoxic, while larger tumors (>1 mm) had minimal hypoxia [55,56]. Tumor hypoxia may induce necrosis or reoxygenation and during cycles of reoxygenation, the cancer cells may acquire a more aggressive phenotype [57]. Additionally, hypoxic tumors may appear dormant with slowly dividing cells or cells resting in G0 which may affect tumor growth and potential response to therapy [58,59,60,61,62,63].

Molecules known to control angiogenesis include HIF1a, SDF1, CXCR4, and VEGF. VEGF is the best-characterized angiogenic factor, with the interaction of CSCR4 and SDF1 leading to the induction of VEGF-mediated angiogenesis, while HIF1 directly regulates VEGF activation [64,65]. De Cuba et al. investigated angiogenesis-related markers and their relation to overall survival (OS), microvessel density, protein expression, and other clinical pathological characteristics in patients with CRC PMs [66]. By promoting angiogenesis and enhancing vascular permeability, VEGF contributes to the development of peritoneal carcinomatosis and is associated with malignant ascites formation with an inverse correlation to OS [67]. This negative correlation between VEGF and OS in CRS is also seen in patients treated with HIPEC. The idea that VEGF negatively affects OS even with treatment is being shown in experimental studies in which VEGF inhibitors enhance drug delivery through lower interstitial blood pressures in tumors [66,68].

Additionally, there is a positive association between high HIF1a expression and favorable resection outcomes which could be explained by increased blood vessel-based oxygen dependence in cells with high HIF1 [66]. HIF1a has also been found to influence metabolic and immune pathways. As tumors become hypoxic, low oxygen levels cause tumor cells to shift from oxidative phosphorylation to glycolysis mediated by HIF1a which leads to upregulation of glucose transporters and genes involved in glycolysis [69,70,71,72]. Hypoxia can also enhance resistance of tumor cells by evading immunosurveillance. HIF1a can regulate genes responsible for tumor immunity under hypoxic conditions [73,74]. Hypoxic regions of tumors have been shown to have high levels of immune-suppressive myeloid-derived suppressor cells (MDSCs), tumor-associated macrophages (TAMs), and regulatory T cells (Tregs). Additionally, with long term exposure to hypoxic conditions, HIF1a has been shown to reduce cytotoxic lymphocytes and inhibit their anti-tumor efficacy [75,76,77]. The relationship of relative hypoxia with reorganization of the tumor microenvironment and promotion of angiogenesis is key to understanding how peritoneal metastases are sustained.

Emerging molecular data also implicate PIPAC in modulating the tumor microenvironment. In colorectal cancer peritoneal metastases, RNA sequencing and proteomics from patients treated with oxaliplatin–PIPAC demonstrated that treatment reduced immunosuppressive signals including hypoxia, IL-10, and TGF-β, while inducing an influx of B and T lymphocytes that organized into tertiary lymphoid structures [78]. Separately, in peritoneal biopsies from patients with pancreatic cancer peritoneal metastasis, histologic regression following PIPAC (assessed by the Peritoneal Regression Grading Score) has been documented, with next-generation sequencing confirming the persistence of detectable KRAS mutations at frequencies similar to primary tumors [79,80]. Additionally, multiplexed mRNA profiling of peritoneal fluid from pancreatic cancer PM patients has identified upregulated tumor-progression markers (e.g., ESRP2, EPCAM, SFN) with prognostic significance, though these studies did not specifically assess hypoxia-related gene signatures [80]. Taken together, these findings suggest that aerosolized intraperitoneal delivery may reshape the peritoneal tumor microenvironment, although direct evidence linking PIPAC to disruption of the hypoxia–angiogenesis axis in pancreatic cancer PM remains to be established.

## 4. Tumor Microenvironment and Immune Landscape

The TME includes not only tumor cells but also nonmalignant cell populations including immune and stromal cells, cancer-associated fibroblasts, endothelial cells and the endocellular matrix [81,82]. The TME of PMs appears to be unique. PMs are the result of considerable interaction between cancer cells, mesothelial cells, and CAFs which secrete extracellular matrix proteins, promoting a favorable environment for PMs [83,84,85,86]. CAFs can derive from mesothelial cells during the formation of PMs and develop during the process of MMT [19].

Takahashi et al. investigated peritoneal ascites in patients with various stages of GC finding the proportions of CD8+ T cells, CD3+/CD56+ NKT-like cells, and CD3-CD56+ NK cells to CD45+ leukocytes were significantly decreased in patients with PM compared to those without PM. Additionally, in patients with PMs, the rates of CD8+ T cells and NKT-like cells inversely correlated with the tumor leukocyte ratio, the proportion of CD19+ B cells was significantly increased, and an M2 macrophage phenotype [40]. Macrophages exist in an M1 anti-tumor/pro-inflammatory phenotype or an M2 pro-tumor/immunosuppressive phenotype and an evaluation of peritoneal fluid found high M2 cells had significantly worse OS [87]. Similarly, in patients with CRC PMs, patients with high intraepithelial CD8+ T cells demonstrated longer disease-free survival and OS [41]. In patients with GC PMs, high tumor CD8+ T cells or a high CD8/CD33 ratio was a better prognosis for OS [42]. In patients with CRC, PMs and synchronous tumors were evaluated; T cells dominated in PMs with evidence of an exhausted phenotype with an increase in programmed cell death protein 1 (PD-1) [88]. In patients who received neoadjuvant chemotherapy, there was a decrease in CD3+ T cells compared to patients who received no neoadjuvant chemotherapy in PMs [89]. Müller et al. studied patients with peritoneal metastasis from colorectal cancer to investigate the effects of systemic therapy. They compared matched peripheral blood samples before and after systemic chemotherapy and identified a decrease in the neutrophil-to-lymphocyte ratio with an increase in circulating T cells in the peripheral blood. In peritoneal fluid, there was a decrease in regulatory T cells [90].

Aronson et al. aimed to characterize the immune TME before and after HIPEC, assessing alterations following treatment. The authors investigated PFS outcomes in patients treated with CRS versus CRS/HIPEC which demonstrated an increase in B cells. There was a depletion of macrophages in the TME which was associated with a higher benefit with CRS HIPEC treatment vs. CRS treatment alone. The subset of patients lacking macrophages and receiving HIPEC were also found to have more favorable OS suggesting a differential effect according to the macrophage status (*p* = 0.054) [43]. In evaluation of peritoneal fluid in patients with PMs, low-grade PMs demonstrated NK cells with an immature phenotype while the high-grade tumors had NK cells with a largely mature phenotype. After CRS/HIPEC, low-grade PMs with an altered NK cell phenotype reverted to normal and exhibited a favorable prognosis [44].

In a pilot study by Fiorentini et al., assessment of peripheral blood in patients with PMs before HIPEC and 30 days post-HIPEC demonstrated significant increases in the number of helper T cells and cytotoxic T cells. It was also interesting in this study that the systemic response was noted to be persistent 30 days post-HIPEC, longer than an acute inflammatory response. The length of time that the systemic immune response persists post-HIPEC as well as the increases in helper and cytotoxic T cells indicates an adaptive immune response post-HIPEC [91]. Tumor-specific immune changes were assessed in mice models injected with murine colon cancer cells to induce peritoneal metastasis. Pathologic assessment of tumor tissue after HIPEC demonstrated more CD8+ T cells, indicating the impact of the immune system in therapeutic effects of HIPEC, as this immune response seems to be related to the control of peritoneal metastasis [41].

Pretreatment systemic inflammatory biomarkers also carry prognostic relevance for PIPAC: in patients with gastrointestinal or ovarian PM undergoing repeated PIPAC, baseline systemic inflammatory biomarkers—including the neutrophil-to-lymphocyte ratio and systemic immune-inflammation index—were independently associated with overall survival and demonstrated moderate discriminatory power for predicting completion of three or more PIPAC cycles, suggesting that the same systemic inflammatory markers implicated in other cytoreductive and intraperitoneal treatment contexts also inform prognosis in the PIPAC setting [92].

Direct comparison of pre-HIPEC tumor tissue and post-HIPEC tumor tissue, although rare in the literature, elucidates important possible mechanisms of action in HIPEC. Dellinger et al. performed a genomic and transcriptomic comparison in epithelial ovarian cancer tissue, with pre-HIPEC tumor samples compared to tumor samples exposed to cisplatin HIPEC in vivo. They also defined good responders to be those with progression-free survival greater than 12 months, and combined clinical outcomes with genomic data to identify possible indicators of good response. Several key findings of note were identified regarding changes induced by HIPEC: upregulation of the inflammatory response and immune pathways, and DNA repair downregulation which was previously discussed. They also demonstrated induced T-cell activation via PD-1 pathways, and also showed that a higher-magnitude PD-1 increase after HIPEC was correlated with longer progression-free survival [48]. PD-1 has long been an area of interest in molecular oncology, and the interaction between HIPEC and PD-1 is actively being explored. PD-1 is a cell surface receptor on T and B cells which works to downregulate the immune response. Though this aids in preventing autoimmune diseases, this can blunt the anti-cancer immune response [93]. The use of PD-1 inhibitors has been shown to be beneficial in many cancers, especially those highly expressing PD-1 [94]. Although PD-1 expression can be variable in peritoneal metastases, anti-PD-1 therapy in murine gastric cancer models led to immune reorganization with increased CD8+ T cells and decreased myeloid-derived suppressor cells within peritoneal metastasis [95,96].

In murine models, it has been shown that combined HIPEC and PD-1 inhibition improved survival when compared to HIPEC alone in patients with metastatic colon cancer [97]. The higher PFS was specifically seen in patients with CD8+ T cells with higher PD-L1 co-expression, regardless of whether the CD8+ T cells were intratumoral (*p* = 0.019) or at the tumor margin (*p* = 0.025) [98]. This could be an important prognostic indicator when choosing which patient to offer combined PDL-1 inhibition therapy and HIPEC [98]. Roth et al. studied CD8+ T cell dynamics in a peritoneal metastasis mouse model, where they found that HIPEC controlled the growth of PM and increased the number of functional granzyme-positive CD8+ T cells within tumors. In cancer cell lines and human organoids that were treated with heated chemotherapy, they noted immunogenic alterations with increased expression of MHC class I molecules as well as priming dendritic cells, which subsequently enhanced effector functions of CD8+ T cells [41].

PD-L1 antibodies are well established and considered first-line for some cancer therapies. Notably, the KEYNOTE 859 established Pembrolizumab and chemotherapy as the treatment regimen for HER-2-negative advanced gastric/esophagogastric junctional cancers [99]. Although this is promising, the establishment of PD-1 therapy in conjunction with HIPEC for metastatic cancer is yet to be proven. Isolated experiences, such as a 2024 case report by Zhou et al., have demonstrated pathological complete response and long-term survival with chemotherapy, HIPEC, and anti-PD-1 immunotherapy in stage IV gastric adenocarcinoma [100]. Isolated case reports are promising, though on a larger scale there is an ongoing trial exploring the benefits of HIPEC in conjunction with the anti-PD-1 antibody (Tirilizumab), SOX chemotherapy and Herceptin [101]. Immune reorganization of the TME plays a key role in the development of peritoneal metastasis and is a promising therapeutic target, especially as ongoing research aims to better characterize the tumor immune microenvironment in different phenotypes of PM.

These findings can be organized by the principal cell populations that shape the peritoneal immune microenvironment. Cancer-associated fibroblasts, frequently derived from mesothelial cells via MMT, secrete extracellular matrix proteins and chemokines that recruit and polarize macrophages while supporting tumor cell adhesion and growth [19,81,82,83,84,85,86]. Macrophages within the peritoneal TME exist along an M1/M2 polarization spectrum; M2-skewed, immunosuppressive macrophages are associated with worse OS, whereas macrophage absence at baseline before HIPEC has been linked to improved progression-free and overall survival, suggesting macrophage status may serve as a predictive biomarker for HIPEC benefit [43,87]. Lymphocyte populations, particularly CD8+ cytotoxic T cells and NK cells, are consistently associated with favorable outcomes when present at high density or in a mature, non-exhausted phenotype, while regulatory T cells and an exhausted, PD-1-high CD8+ phenotype track with disease progression and reduced treatment response [40,41,42,44,88,89,90]. These cell-type-specific patterns are not independent however; CAF-derived signaling shapes macrophage polarization, and absent baseline macrophage levels appear to potentiate CD8+ T-cell-mediated anti-tumor activity after HIPEC, underscoring that the peritoneal TME functions as a possible interdependent cellular network rather than a set of isolated compartments [43,82].

## 5. Future Directions

Patient survival with PMs remains poor despite advancements in systemic chemotherapy. Patients with PM have worse survival than other metastatic disease sites such as the liver or lung [6]. It is believed that due to limited blood supply to PMs, these tumors have a decreased response to systemic chemotherapy. Additionally, the role of the immune component of the TME in cancer is increasingly recognized. IP therapies such as HIPEC have been used for decades to treat PMs. However, the utility and efficacy of HIPEC has been called into question [5]. Although there have been significant advances in systemic chemotherapy, marginal advances have been made with IP chemotherapy in terms of drug development and targeted therapies. Therefore, IP chemotherapy remains a rapidly evolving therapeutic modality with significant potential for clinical advancement with many ongoing clinical trials assessing the utility of IP chemotherapy (Table 2).

The use of immunotherapy in conjunction with HIPEC is an active area of clinical interest with potential for future advancement. There are ongoing clinical trials exploring the benefits of combined immunotherapy and HIPEC. For instance, the use of PD-1 inhibitors, Camrelizumab and Sintilimab, in conjunction with HIPEC is an area of interest for patients with advanced gastric cancer [NCT06213519]. As the landscape of intraperitoneal chemotherapy continues to advance, understanding molecular oncology and the tumor microenvironment will be critical to improve clinical outcomes. Modern translational techniques aid in this effort to better understand tumor microenvironment characteristics and their relationship to treatment response. High-resolution techniques, such as spatial transcriptomics and proteomics, can allow for novel in-depth insights into tumor immune microenvironment spatial organization and response to treatment. Cytoreductive surgery for CRC PMs has been demonstrated to improve survival yet carries significant potential of post-operative morbidity [5]. Given the potential risk of post-operative complications and resulting morbidity in patients with metastatic disease, selection is paramount, targeting patients who will truly benefit from the highly invasive interventions. Optimization of patient selection remains a central focus of ongoing research. Current selection criteria emphasize clinical parameters such as patient age, performance status, disease histology, biology and burden of disease with the ability to achieve a complete resection [102].

Beyond anatomic resectability, emerging evidence suggests that the molecular subtype of the primary tumor should be factored into patient selection. The immunologic composition of the TME, particularly the ratio of CD8+ cytotoxic T cells to immunosuppresive cell populations such as M2 macrophages and Tregs, may serve as a pre-operative predictor of CRS-HIPEC response. Given that macrophage depletion within the TME after HIPEC has been associated with survival benefit, and that high intraepithelial CD8+ T-cell density correlates with improved disease-free survival in CRC peritoneal metastases, integrating immune profiling of biopsy specimens into pre-operative workup may be a logical next step in refining patient selection [41,43].

Guidelines are evolving to further incorporate tumor-specific molecular markers into the pre-operative workup. In metastatic colon cancer for instance, mutations in BRAF and RAS are utilized as biomarkers to determine cytoreduction eligibility. Notably, RAS has been shown to be a marker of decreased survival and increased disease recurrence after CRS and HIPEC [103]. Further research is ongoing, exploring other biomarkers as potential tools for treatment guidance. Studies have shown that elevated VEGF expression has been associated with worse OS [66]. Refining patient selection through tumor biology and molecular profiling remains an expanding field which will allow clinicians to provide a patient-personalized treatment approach. Furthermore, establishing associations between biochemical markers and tumor behavior may also aid in predicting response. Across metastatic cancers of various origins, studies have established prognostic markers that are promising. In appendiceal-origin peritoneal carcinomatosis for example, carcinoembryonic antigen (CEA) and carbohydrate antigen 19-9 (CA 19-9) have been explored as predictors of response [104,105]. Elevated pre-operative CA 19-9 has similarly been linked to poorer PFS. Additionally, there is ongoing research to study the utility of circulating tumor DNA as a possible marker for response monitoring, although data still remains conflicting and larger multicenter cohorts across various cancer types are needed to establish its true prognostic value [106,107,108].

Beyond biomarker-based selection, pathway-specific therapeutic vulnerabilities are also being explored; Dahlmann et al. demonstrated that PARP inhibition was effective in patient-derived xenograft models of peritoneal metastasis harboring impaired DNA repair capacity, suggesting that the DNA repair pathway alterations induced by IP chemotherapy (Section 2.3) may represent an exploitable therapeutic target rather than solely a marker of treatment response [109]. Furthermore, an emerging and particularly promising avenue is the application of patient-derived organoids (PDOs) for individualized sensitivity testing prior to CRS and IP chemotherapy. This organoid platform allows for ex vivo recapitulation of the tumor’s molecular architecture and can be used to screen for sensitivity to chemotherapeutic agents. This allows for a patient-tailored approach to sensitivity screening for chemotherapy, including intraperitoneally delivered variants, before committing to the significant physiologic burden of CRS-HIPEC. The inclusion of organoid sensitivity testing as a stratification tool in the Hi-STEP1 trial (NCT05652348) reflects a promising future direction in further optimizing patient selection. If validated prospectively, PDO-based sensitivity data could allow clinicians to meaningfully refine patient selection beyond static molecular markers, capturing functional chemoresistance that is otherwise unknown with conventional testing.

Circulating tumor DNA (ctDNA) also holds potential as a minimally invasive marker for response monitoring in the peri-operative period. Serial ctDNA sampling before and after CRS-HIPEC can allow for early identification of patients with residual molecular disease, prompting proactive treatment regimen adjustments. Larger multicenter prospective studies correlating ctDNA dynamics with long-term oncologic outcomes and establishing ctDNA utility based on primary pathology are needed to establish its true clinical utility.

## 6. Conclusions

Peritoneal metastases are a challenging manifestation of advanced malignancy, in part because the peritoneal cavity represents a distinct biological and therapeutic environment. Although CRS and IP have improved outcomes for selected patients, treatment responses remain heterogeneous and difficult to predict. Developing data suggest that PMs are not simply an anatomic pattern of spread, but rather tumors shaped by unique molecular alterations, metabolic adaptations, angiogenic signaling, and immune microenvironmental pressures.

Intraperitoneal chemotherapy, particularly HIPEC, appears to induce measurable changes in this environment, including alterations in gene expression, DNA repair pathways, immune-cell composition, and PD-1/PD-L1 signaling. At the same time, angiogenic mediators such as VEGF and HIF1α continue to influence tumor progression, treatment response, and patient outcomes. These findings support the need to move beyond traditional selection criteria alone and toward a more biologically informed and patient-personalized approach to PM management.

Future studies should focus on validating molecular and immune biomarkers that can better identify patients more likely to benefit from CRS and IP chemotherapy. At the same time, the potential synergy between IP and immunotherapy represents an important and growing area of ongoing investigation. A deeper understanding of the tumor microenvironment in PMs may ultimately inform refined patient selection, guide combination strategies, and improve outcomes for patient populations that face limited therapeutic options.

## Figures and Tables

**Table 1:** Intraperitoneal chemotherapy delivery methods.

IP Chemotherapy Delivery Method	Acronym	Methodology
Heated Intraperitoneal Chemotherapy	HIPEC	Intraperitoneal chemotherapy heated to 41–43 °C
Normothermic Intraperitoneal Chemotherapy/Early Post-Operative Chemotherapy	NIPEC/EPIC	IP chemotherapy delivered at normothermic conditions often through indwelling intra-abdominal catheters
Pressurized Intraperitoneal Aerosolized Chemotherapy	PIPAC	Laparoscopic delivery of intra-abdominal chemotherapy as an aerosol utilizing capnoperitoneum

**Table 2:** Ongoing clinical trials of intraperitoneal chemotherapy for gastrointestinal malignancies.

NCT Number	Title	Acronym	Condition	Phase	Status
HIPEC TRIALS					
NCT04779554	Flat Dose vs. Weight-based IP Chemotherapy for CRS/HIPEC		Peritoneal carcinomatosis	2	Recruiting
NCT07282834	Heated Versus Aerosol-based Laparoscopic Chemotherapy for Cancer That Has Spread to the Peritoneum (Abdominal Lining)	Charlie-2	Peritoneal carcinomatosis	2	Recruiting
NCT05250648	Clinical Trial on HIPEC With Mitomycin C in Colon Cancer Peritoneal Metastases	GECOP-MMC	Colon cancer	4	Recruiting
NCT04107077	Phase II Study of the Effects of Laparoscopic HIPEC in Patients With Advanced Gastric Cancer		Gastric cancer	2	Recruiting
NCT07493421	To Evaluate the Feasibility and Safety of Combining Surgery (Pancreatectomy and Cytoreduction) With HIPEC for Treating Pancreatic Cancer With Peritoneal Involvement.		Pancreatic cancer	N/A	Recruiting
NCT05652348	Response Prediction of Hyperthermic Intraperitoneal Chemotherapy in Gastrointestinal Cancer	Hi-STEP1	Gastric cancer, colon cancer	N/A	Recruiting
NCT04847063	Individual Response to HIPEC Treatment of Peritoneal Carcinomatosis From Peritoneal Mesothelioma or Atypical Mesothelial Proliferation or From Ovarian, Colorectal, or Appendiceal Histologies		Mesothelioma; ovarian, appendiceal, colorectal cancer	1	Recruiting
PIPAC/OTHER TRIALS					
NCT05395910	PIPAC and ePIPAC With Paclitaxel In Patients With Peritoneal Carcinomatosis		Peritoneal carcinomatosis	1	Recruiting
NCT06367270	The Application of PIPAC for Peritoneal Surface Malignancies	PIPAC	Peritoneal carcinomatosis	2	Recruiting
NCT03280511	Adjuvant PIPAC in Resected High Risk Colon Cancer Patients		Colorectal cancer	2	Recruiting
NCT04595929	Oncological Benefits of Pressured Intraperitoneal Aerosol Chemotherapy (PIPAC) in Patients With T3-4 Gastric Cancer	GASPACCO	Gastric cancer	3	Recruiting
NCT05913674	Technical Feasibility of Modified EPIC (mEPIC)		Appendiceal and colorectal cancer	2	Recruiting
NCT07001748	Testing the Addition of Paclitaxel Administered Into the Abdominal Cavity Combined With Chemotherapy for Patients With Gastric Cancer Spread to the Abdominal Cavity	STOPGAP II	Gastric cancer	2/3	Recruiting
NCT07030283	IP Paclitaxel With NALIRIFOX for Pancreatic Ductal Adenocarcinoma With Peritoneal Carcinomatosis		Pancreatic cancer	1	Not yet recruiting
NCT04329494	PIPAC for the Treatment of Peritoneal Carcinomatosis in Patients With Ovarian, Uterine, Appendiceal, Colorectal, or Gastric Cancer		Ovarian, uterine, appendiceal, colorectal, or gastric Cancer	1	Recruiting
NCT06784765	Preventive Use of PIPAC in Locally Advanced Gastric Cancer.		Gastric cancer	N/A	Recruiting

PIPAC, pressurized aerosolized intraperitoneal chemotherapy; ePIPAC, Electrostatic PIPAC; IP, intraperitoneal; CRS, cytoreductive surgery; HIPEC, heated intraperitoneal chemotherapy; EPIC, Early Post-Operative Intraperitoneal Chemotherapy.

## Data Availability

No new data was created during this study.
